# Distinctive serum protein profiles involving abundant proteins in lung cancer patients based upon antibody microarray analysis

**DOI:** 10.1186/1471-2407-5-110

**Published:** 2005-08-23

**Authors:** Wei-Min Gao, Rork Kuick, Randal P Orchekowski, David E Misek, Ji Qiu, Alissa K Greenberg, William N Rom, Dean E Brenner, Gilbert S Omenn, Brian B Haab, Samir M Hanash

**Affiliations:** 1 Department of Internal Medicine, University of Michigan, Ann Arbor, MI 48109, USA; 2 Department of Critical Care Medicine, Safar Center for Resuscitation Research, University of Pittsburgh, Pittsburgh, PA 15260, USA; 3 Department of Pediatrics, University of Michigan, Ann Arbor, MI 48109, USA; 4 Van Andel Research Institute, Grand Rapids, MI 49503, USA; 5 Division of Pulmonary and Critical Care Medicine, NYU Cancer Institute, NYU School of Medicine NY, NY 10016, USA; 6 Fred Hutchinson Cancer Research Center, Seattle, WA 98109, USA

## Abstract

**Background:**

Cancer serum protein profiling by mass spectrometry has uncovered mass profiles that are potentially diagnostic for several common types of cancer. However, direct mass spectrometric profiling has a limited dynamic range and difficulties in providing the identification of the distinctive proteins. We hypothesized that distinctive profiles may result from the differential expression of relatively abundant serum proteins associated with the host response.

**Methods:**

Eighty-four antibodies, targeting a wide range of serum proteins, were spotted onto nitrocellulose-coated microscope slides. The abundances of the corresponding proteins were measured in 80 serum samples, from 24 newly diagnosed subjects with lung cancer, 24 healthy controls, and 32 subjects with chronic obstructive pulmonary disease (COPD). Two-color rolling-circle amplification was used to measure protein abundance.

**Results:**

Seven of the 84 antibodies gave a significant difference (p < 0.01) for the lung cancer patients as compared to healthy controls, as well as compared to COPD patients. Proteins that exhibited higher abundances in the lung cancer samples relative to the control samples included C-reactive protein (CRP; a 13.3 fold increase), serum amyloid A (SAA; a 2.0 fold increase), mucin 1 and α-1-antitrypsin (1.4 fold increases). The increased expression levels of CRP and SAA were validated by Western blot analysis. Leave-one-out cross-validation was used to construct Diagonal Linear Discriminant Analysis (DLDA) classifiers. At a cutoff where all 56 of the non-tumor samples were correctly classified, 15/24 lung tumor patient sera were correctly classified.

**Conclusion:**

Our results suggest that a distinctive serum protein profile involving abundant proteins may be observed in lung cancer patients relative to healthy subjects or patients with chronic disease and may have utility as part of strategies for detecting lung cancer.

## Background

Lung cancer remains the leading cause of cancer mortality in the United States for both men and women [1,2]. Despite significant advances in understanding its biology and causes, the overall incidence of lung cancer is increasing, and improvements in outcome are not apparent [3]. As treatment is efficacious only for those patients who are diagnosed sufficiently early in the disease process, a significant reduction in patient mortality may result from earlier detection of lung cancer, including combinations of biomarkers with spiral CT imaging [2].

Identification of protein biomarkers in blood or serum may have utility for noninvasive disease detection and classification. Biomarker identification would be greatly enhanced by methodological improvements in protein detection. Direct serum protein profiling by matrix assisted laser desorption ionization (MALDI) mass spectrometry [4,5] has uncovered distinct mass profiles in several common types of cancer. However, the direct profiling of complex protein mixtures by MALDI has difficulties in providing the identification of the distinctive proteins. Further, given the limited dynamic range of MALDI, it is likely that distinctive features observed in serum with this approach represent relatively abundant proteins.

An alternative to mass spectrometry for protein profiling is the use of antibody microarrays. The field of protein microarrays currently encompasses applications that include profiling of serum and tissues from cancer patients [6,7], autoimmune diagnostics [8], protein interaction screening [9-12], as well as antibody-based detection of multiple antigens [13-17]. Recent increases in sensitivity and quantitative reproducibility has extended the utility of antibody microarrays [18,19]. In particular, direct multicolor labeling with rolling-circle amplification (RCA) has enabled enhanced sensitivity and reproducible measurements of low-abundance proteins, as compared to other direct or indirect labeling detection methods [20,21]. The strategy behind the two-color RCA detection is that two different protein samples can be labeled, respectively, with either biotin or digoxigenin, then both samples are co-hybridized to the antibody arrays. The bound proteins are then detected and individually quantitated, using RCA (and Cy3) to amplify fluorescence signals emanating from the bound biotin-labeled proteins, and RCA (and Cy5) to amplify signals from bound digoxigenin-labeled proteins. We have previously reported that, in comparison with either direct or indirect labeling detection, two-color RCA produced up to 30-fold higher fluorescence intensity measurements, enabling the reproducible measurements of lower abundance proteins in serum [20]. Importantly, we have been able to ascertain reproducible small expression differences between 2 different samples.

In the present study, we have utilized the RCA methodology and a panel of 84 antibodies to analyze the relative abundance of multiple proteins in sera from 24 newly diagnosed patients with lung cancer, 24 healthy controls, and 32 patients with chronic obstructive pulmonary disease (COPD). We have identified a distinctive serum protein profile for patients with lung cancers.

## Methods

### Serum samples

Serum samples were obtained following informed consent from 80 individuals, including 24 newly diagnosed lung cancer patients, 24 healthy subjects without a prior history of cancer, and 32 patients with COPD. All samples were collected under protocols approved by the local Institutional Review Board (IRB). The sera from lung cancer patients and healthy controls were collected through the Early Detection Research Network (EDRN) program at the University of Michigan. The sera from COPD patients were collected through the EDRN at New York University. All samples were stored frozen at -80°C prior to analysis.

### Construction of antibody microarrays

The antibodies used in the preparation of the microarrays were purchased from various sources (a complete list and further information on each antibody is available online [22]). The 84 antibodies targeted 80 different proteins, present at a broad range of concentrations in serum, that could have levels associated with lung cancer, including acute phase reactants, proteases and protease inhibitors, immune system proteins, glycoproteins, extracellular matrix proteins, and cytokines. Microarray preparation was performed as described previously [20,23]. Briefly, samples (20 μl each) of 100–2000 μg/ml antibody solutions in PBS were prepared in polypropylene 384-well microtiter plates (MJ Research). Small amounts of each antibody solution were transferred to the surface of nitrocellulose-coated microscope slides (PATH slides, Gentel Biosurfaces) using a piezoelectric non-contact spotter (Biochip Arrayer, PerkinElmer Life Sciences). Twelve identical arrays were printed on each of seven slides; each array consisted of 96 antibodies or control proteins ("baits") printed in triplicate to form an 18 by 16 array of dots.

### Serum labeling

An aliquot (1 μl) from each of 80 serum samples was labeled with N-hydroxysuccinimide (NHS)-Digoxigenin (Molecular Probes), and a second aliquot (1 μl) was labeled with NHS-biotin (Molecular Probes). Each 1 μl serum aliquot was diluted with 14 μl PBS containing 500 μM NHS-biotin or NHS-Digoxigenin. After the reactions had proceeded for 1 h on ice, 5 μl of 1 M Tris-HCl (pH 7.5) was added to each tube to quench the reactions, then the solutions were incubated on ice for an additional 20 min. Non-reactive dye molecules were removed by passing each solution through a size-exclusion chromatography spin column (Bio-Spin P6, Bio-Rad) with a molecular weight cutoff of 6 kDa. The digoxigenin-labeled samples were pooled, then distributed equally among the biotin-labeled samples. 4 μl of Tris-buffered saline (TBS) containing Super Block (Pierce), 1% Brij-35, and 1% Tween-20 was added to each sample, after which the total volume of each sample was adjusted to 40 μl with TBS.

### Processing of antibody microarrays

Each of the 12 arrays on a slide was circumscribed with a wax border to segregate the arrays from each other. The slides were rinsed twice in PBS with 0.5% Tween-20 (PBST0.5) and then blocked 1 h at 4°C in PBS containing 0.1% Tween-20 (PBST0.1), 0.3% CHAPS, and 1% BSA. After the arrays were briefly rinsed twice with PBST0.5 and dried by centrifugation, 40 μl of each labeled serum sample mix was incubated on an array with gentle rocking at room temp for 1 h. The three groups of samples were arranged so as to balance the types of samples on each slide, as shown in a supplementary table. The arrays were rinsed in PBST0.1, briefly washed three times in PBST0.1, then dried by centrifugation. Mouse monoclonal anti-Biotin (Jackson ImmunoResearch) was covalently conjugated to a 20-base oligonucleotide (primer 1) as previously described [20]. Molecular Staging (New Haven, CT) kindly provided the other reagents necessary for RCA detection. These included a mouse monoclonal anti-Digoxigenin (Roche) antibody conjugated to a different 20-base oligonucleotide (primer 4.2), an 81-base circular DNA (circle 1) with a portion complementary to primer 1, and an 80-base circular DNA (circle 4.2) with a portion complementary to primer 4.2. The sequences of the primers, circles and decorators can be found in the supplementary information for Zhou et al. [20]. The microarrays were incubated for 1 h at room temp in PBST0.1 containing 1 mM EDTA, 5 mg/ml BSA, 75 nM circle 1, 75 nM circle 4.2, 1.0 μg/ml primer 1-conjugated anti-biotin, and 1.0 μg/ml primer 4.2-conjugated anti-Digoxigenin. The arrays were rinsed briefly in PBST0.1 then washed at room temp with gentle rocking three times for 3 min each in PBST0.1, after which they were incubated in 1X Tango buffer (Fermentas, Hanover, MD) containing Phi29 DNA polymerase (New England Biolabs), 0.1% Tween-20 and 0.4 mM dNTPs at 37°C for 30 min. Following a brief rinse in 2X SSC with 0.1% Tween-20 (SSCT0.1), the arrays were washed three times for 3 min each at room temperature with gentle rocking in 2X SSCT0.1, then dried by centrifugation. The arrays were incubated for 1 h (37°C) in 2X SSCT0.1 containing 0.5 mg/ml herring sperm DNA, Cy3-labeled 18-bp oligonucleotide complementary to the repeating DNA strand from primer 1 and a Cy5-labeled 22-bp oligonucleotide complementary to the repeating DNA strand from primer 4.2, each at 0.1 mM. The arrays were briefly rinsed in 2X SSCT0.1, washed three times for 3 min each at room temperature in 2X SSCT0.1, dried by centrifugation, then scanned (ScanArray, PerkinElmer Life Sciences).

### Analysis

The Cy3 and Cy5 fluorescence was quantified using GenePix software (Axon Instruments). Of the total of 24192 dots, 206 were excluded as having defects by visually inspecting the images without reference to the quantitative data, with the most common cause of the defect being overlapping dots. The resultant ".gpr" files for each array were parsed to create a spreadsheet of the raw data, available as a supplement [22]. We took the negative of the base-2 logarithm of the "median of ratios" computed by the software, and averaged the triplicate measures for each bait, not including the excluded dots. This gave the average of the log-ratio of the sample (Cy3) to the standard pool (Cy5), hereafter referred to as the values.

We first performed a normalization in which the median value for each array was subtracted from all the values for that sample. Some antibodies displayed biases in favor of either the Cy3 or Cy5 channel, or showed large differences between groups. Consequently, we selected a subset of 48 antibodies that did not have large differences between groups, and had small within-group standard deviations in order to perform a normalization that would be less affected by antibodies with variable data or channel biases. We computed the average of the raw values for each antibody using the 80 arrays, and normalized the individual slides to this standard. For each slide, the median of the 48 differences for the array minus the corresponding values on the standard was subtracted from the array, subtraction being used rather than division because the values were already log-transformed. The averaged raw and normalized data are available as supplemental information [22].

### Western blot analysis

We used Western blots to analyze the level of C-reactive protein (CRP) and serum-amyloid A (SAA) in sera of eight selected lung cancer patients and eight healthy controls. Subsequently, in order to validate our findings, we also analyzed the CRP and SAA levels in an independent set of 30 additional lung cancer patients and 30 additional healthy controls. Briefly, 5 μl of serum (from each patient) was resolved by 15% SDS-PAGE, and then transferred to a PVDF membrane. Following incubation in blocking buffer (PBST0.1 containing 2% nonfat dry milk (Bio-Rad)) for 2 h, the membrane was hybridized in blocking buffer containing either anti-CRP or anti-SAA mouse monoclonal antibodies at 0.5 μg/ml and 0.25 μg/ml for 1 h. The membrane was then washed and incubated with a horseradish peroxidase-conjugated sheep anti-mouse IgG (Amersham) at a 1:1000 dilution for 1 h. After washing, the membrane was briefly incubated in ECL (Enhanced Chemiluminescence, Amersham), then exposed to imaging film (Amersham). Integrated intensity measurements were made of the respective bands and the measurements were further analyzed statistically.

## Results

**Figure 1:**
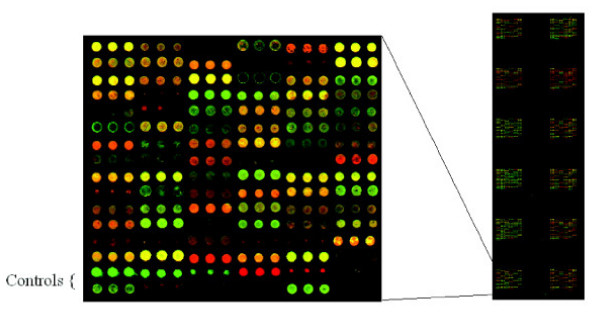
Scanned fluorescence image of an antibody microarray detected by two-color RCA. 96 baits including 84 antibodies were spotted onto microscope slides coated with nitrocellulose. 12 identical arrays were printed on each of seven slides. Each antibody was printed in triplicate on each array in order to form an 18 by 16 array of dots. A test sample labeled with biotin and a pooled reference sample labeled with digoxigenin were co-incubated on the microarray, and bound proteins from both samples were detected by RCA. The microarray was scanned for Cy3 fluorescence (from the test sample) and Cy5 fluorescence (from the reference sample).

Using microarrays containing 84 antibodies printed in triplicate on slides, we measured the amount of target protein bound from 80 individual sera, with each sample being compared to a pooled reference sample (consisting of a mixture of all of the sera) in a two-color assay. Figure 1 shows a representative image of antibody arrays from one slide. Eighty arrays with 24 sera from lung cancer patients, 24 normal sera, or 32 sera from patients with COPD were analyzed. The values determined were the normalized average of base-2 logarithms of the intensity arising from the individual sample divided by the intensity arising from the pooled sample, which was measured as Cy3 and Cy5 fluorescence, respectively. Values from triplicate antibody dots from the same array were quite reproducible, with average standard deviations of 0.14, corresponding to approximately 10% variation in the ratios.

**Figure 2:**
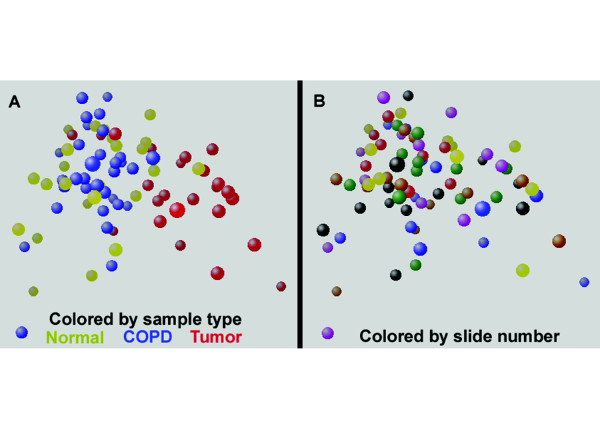
The first 3 principal components from normalized log-base-2 ratios of sample to reference pool intensities, using all 84 antibodies. The full 3-dimensional figures that can be rotated are available in the supplementary materials. In A, normal, COPD and lung cancer patients are marked with yellow, blue and red, respectively. The first three principal components account for 43% of the variance. In B, seven slides are marked separately with blue, black, yellow, green, purple, brown and red.

Figure 2 depicts the first three principal components obtained using all 84 antibodies. While lung cancer patients were largely separated from the other two groups of patients, there was no clear separation between COPD and normal. This completely unsupervised view of the data indicates that the distinction between lung tumor patients' sera and the two other groups of sera was likely the largest source of variation in the data set (Figure 2A). The somewhat outlying samples were not associated with a particular microarray slide (Figure 2B) or brightness of the signals for either fluorescence. The first principal component was most highly correlated with C-reactive protein (CRP) and serum amyloid A (SAA).

**Figure 3:**
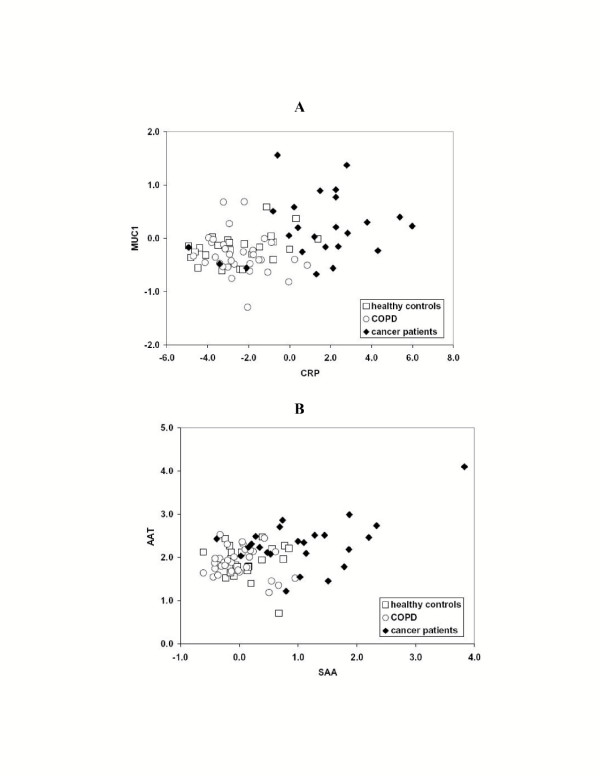
Two-dimensional plots of normalized log-base-2 ratios of sample to reference pool intensities for CRP and MUC1, and SAA and AAT.

In order to determine which antibodies distinguished sera of lung tumor patients from the other sera, we fit a 1-way analysis of variance model to the three groups of samples. Cancer patient sera gave significantly different mean values for 7/84 antibodies when compared to normal sera, and for 8/84 of the antibodies when compared to the COPD sera (both at p < 0.01). The 7 antibodies that yielded differences in the abundance of their corresponding proteins between tumor and normal sera were common to the group of 8 antibodies that yielded differences in the abundance of their corresponding proteins between tumor and COPD sera. The additional protein identified by the COPD comparison is troponin 1. We found increased levels of CRP, SAA, α-1-antitrypsin (AAT) by two distinct antibodies, and MUC1, and decreased levels of transferrin and gelsolin, in lung cancer sera (Table 1). Results obtained for the entire set of antibodies are available as supplemental data [22]. To assess the significance of these findings, we randomly permutated the sample labels 1000 times and performed the identical analysis on each resulting data set. On average this yielded only 0.1 antibodies for which the tumor samples were increased or decreased (at p < 0.1) compared to both other groups, with 1 or more significant antibody found in only 8.1% of the permuted data sets. Therefore, it is very unlikely that the occurrence of differences in levels of proteins for the 7 antibodies observed in the actual data is due to chance. The correlation within the group of lung cancer patients between the CRP, SAA, AAT, MUC1, transferrin and gelsolin data values are summarized in Table 2, and the two-dimensional log-scale plots for CRP and MUC1, and SAA and AAT are shown in Figure 3. The expression levels of CRP, SAA and AAT but not MUC1 were correlated with each other (r > 0.4, p < 0.05). The two AAT measurements, each derived from a different antibody, were significantly correlated (r = 0.72, p < 0.001).

**Figure 4:**
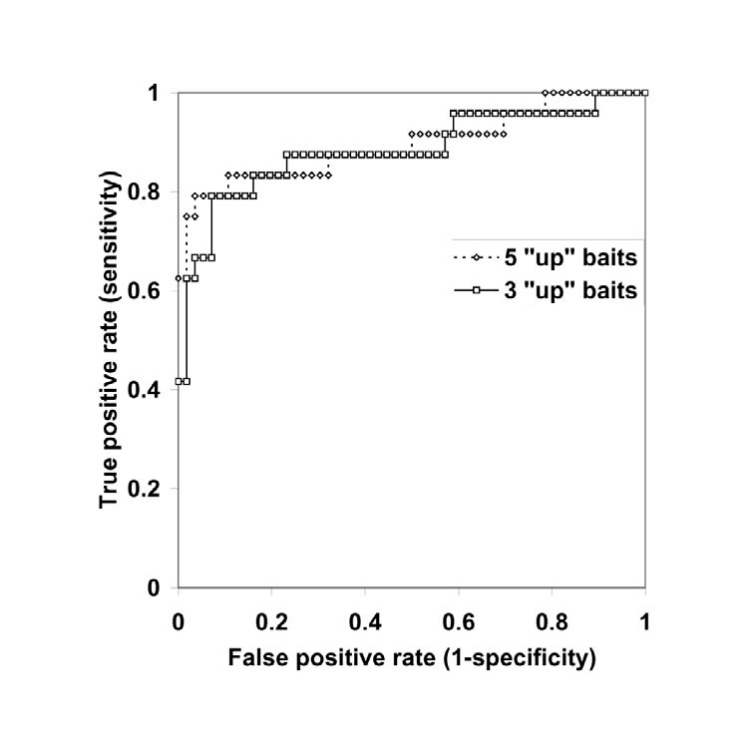
Receiver Operating Characteristic (ROC) curves from leave-one-out validation of a Diagonal Linear Discriminant Analysis classifier using the best 3 (or 5) antibodies. Both the antibodies selected and the discriminant function were based solely on the remaining 79 samples.

We performed a leave-one-out validation of a Diagonal Linear Discriminant Analysis (DLDA) classifier that discriminates tumor vs. non-tumor samples [23]. We left out one sample at a time, then used the remaining 79 samples to select the 5 antibodies with values increased in tumor patient samples according to the p-values for 2-sample T-tests of tumor vs. non-tumor samples, and constructed the resulting discriminant function based on the 79 samples. When using all of the data CRP, SAA, MUC1, and 2 AAT antibodies would be selected as the top antibodies, in that order. The value of this function was then computed for the left out sample. Figure 4 shows the resulting Receiver Operating Characteristic (ROC) curve that was obtained. The calculations were also repeated using only the best 3 antibodies. Using 5 antibodies, the correct classification of all 56 of the non-tumor samples was associated with the correct classification of 15 of 24 cancer patient sera. We obtained the same result with a different classifier that used majority voting among the 5 closest neighboring samples, where the distances were computed after scaling each antibody's values by the pooled estimate of the standard deviation (in analogy to DLDA). Analogous results from cross-validating this simpler classifier using only the 3 best antibodies correctly classified 17 of 24 cancer patient sera, while misclassifiying 4 of 56 non-tumor samples, which also corresponds approximately to a point on the ROC curve for the DLDA classifier when it used 3 antibodies. This illustrates that the results obtained with DLDA classifiers were not particularly better than could be obtained with other simple methods.

**Figure 5:**
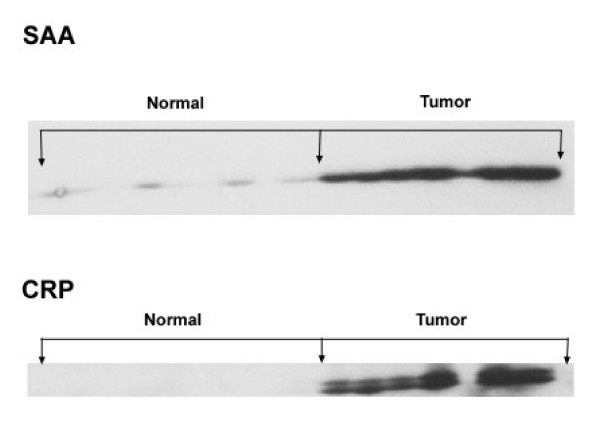
SDS-PAGE Western blot analysis of CRP and SAA. CRP and SAA levels in sera of eight lung cancer patients and eight healthy controls were analyzed. The sera chosen were those that gave extremely high or low values for the corresponding assay on the antibody microarrays.

**Figure 6:**
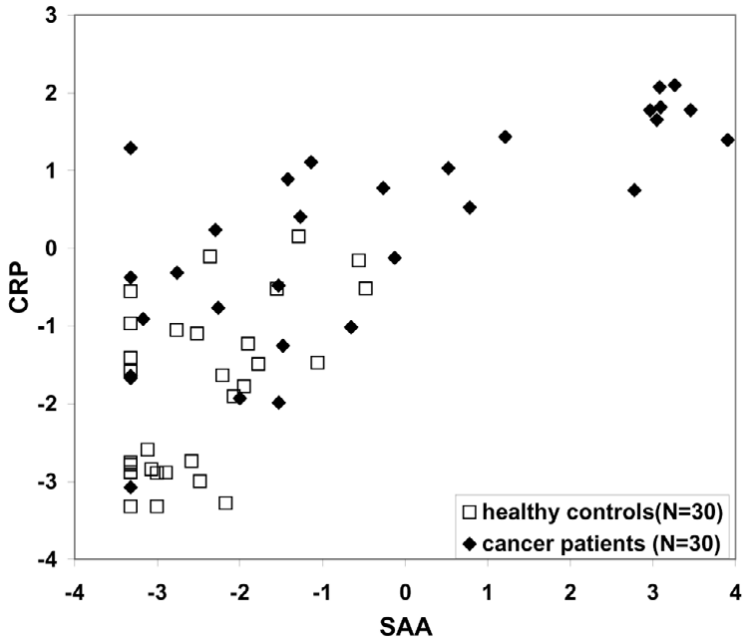
A scatter plot of integrated intensity measurements derived from western blots of an independent set of sera from 30 additional lung cancer patients and 30 additional healthy controls, probed for SAA and CRP. Values are base two logarithms of the relative band intensities after adding 0.1 to each value (to force values to be greater than 0).

CRP and SAA were selected for Western blot analysis in order to validate the specificity of antibody microarrays. Eight lung cancer sera and 8 normal sera were resolved by SDS-PAGE, then transferred to PVDF membranes. The membranes were probed with anti-CRP or anti-SAA antibodies. As shown in Figure 5, all of the sera from patients with lung cancer showed much higher levels of CRP and SAA compared to the sera from healthy controls. Subsequently, in order to validate our findings, we also analyzed the CRP and SAA levels in an independent set of 30 additional lung cancer patients and 30 additional healthy controls. Integrated intensity measurements were made of the respective bands and the measurements were further analyzed statistically. The distribution of integrated intensity measurement values obtained from the two groups of samples for both assays are shown in Figure 6. The number of tumor samples with values greater than the largest value for normal samples was 17/30 for CRP (p = 3.1 × 10^-7^) and 13/30 for SAA (p = 2.3 × 10^-5^).

## Discussion

Four proteins were found to be more abundant in the lung cancer samples than those of the controls, namely CRP (13.3 fold), SAA (2.0 fold), AAT (1.4 fold) and MUC1 (1.4 fold). There were no significant protein expression differences observed in serum between the various lung cancer subtypes examined (adenocarcinoma, squamous and small cell carcinomas: data not shown). The significant increases in CRP and SAA protein levels found in the serum of lung cancer patients by protein microarray were confirmed by immunoassay. The increased levels of AAT in lung cancer patient sera (1.4 fold) were observed using two different antibodies, each obtained from a separate source.

The pattern of increased abundances of CRP, SAA, AAT and MUC1 in lung cancer patient sera that were observed in our microarray-based study is concordant with previous studies of individual proteins. An increased C-reactive protein level is part of the acute-phase response to most forms of inflammation, infection, tissue damage, and malignant neoplasia [25-27]. CRP [Uniprot PO2741] forms homopentamers (pentaxins); it promotes phagocytosis and complement fixation through calcium-dependent binding (two per 23 kDa subunit) to phosphorylcholine. CRP also interacts with DNA and histones to scavenge nuclear material from damaged circulating cells. The expression of CRP is induced by IL-1 and IL-6. While CRP itself is likely not useful as a single assay, it may have clinical utility as part of a panel of diagnostic biomarkers, especially in evaluating results from spiral CT imaging [2]. CRP is mainly expressed in hepatocytes; cytokines, especially interleukin-6, induce the expression and release of CRP [28,29]. CRP has been suggested as a useful prognostic indicator in esophageal carcinoma [30]. Studies also showed that CRP was an independent determinant of survival in non-small-cell lung cancer [31] and could be useful in the initial evaluation of patients with small cell lung cancer and in monitoring response to therapy [32].

Serum amyloid A [Uniprot PO2735] is an acute-phase protein that occurs in various isoforms in a molecular mass range of 11–14 kDa. SAA is produced by hepatocytes [33], secreted into serum and rapidly binds to high-density lipoprotein, with 90% occurring in the bound form [34]. SAA occurs at low levels in sera of healthy individuals [35]. Patients with neoplastic disease, including lung [36], renal [37], colorectal [38], prostate [39] and nasopharyngeal cancers [40] exhibit a dramatic elevation of serum SAA. However, SAA is not a cancer-specific marker *per se*. Its elevation in serum has been reported also in association with trauma, infection, inflammation, rheumatoid arthritis, and amyloidosis [41]. A study of 621 subjects with cancer found substantial increases of SAA levels in >95% (281 of 289) of patients with metastatic solid tumors, all myelocytic leukemia patients and all advanced lymphoma patients [42]. Interestingly, SAA was not elevated in the group of 32 COPD patients included in this study, suggesting a potential utility of SAA in distinguishing between the two conditions possibly due to a different cytokine profile between the two groups.

α-1-antitrypsin [A1AT/SERPINA1, Uniprot PO1009] is a secretory glycoprotein of molecular weight 44 kDa produced in the liver. It neutralizes the effects of proteases in several organ systems, mainly in the lung. The major physiological role of AAT in the lung is to bind and inhibit elastase released from leucocytes in the lower respiratory tract, thereby preventing the destruction of lung tissue [43,44]. The normal range of serum or plasma AAT concentrations is 1200–2000 mg/L, with large increases in inflammatory conditions, infections, cancer, liver disease, or pregnancy [43]. It was previously reported that the serum concentration of AAT increased with tumor growth and could be utilized following tumor resection as an indicator of relapse [45,46]. The prognostic significance of AAT expression in lung adenocarcinomas has been evaluated using immunohistochemistry [47]; strongly AAT-positive cases had a worse prognosis than weak-to-moderately AAT-positive or AAT-negative cases, suggesting that increased AAT expression in lung adenocarcinoma patients may be a prognostic indicator. The biological basis for the association of acute-phase proteins, including CRP, SAA, and AAT, with lung cancer remains largely unknown. The correlation between CRP, SAA, and AAT levels was significant (r > 0.4), likely reflecting a host response. Significantly higher levels occur in patients with metastatic disease compared to patients with limited disease [48].

We found serum MUC1 levels to be modestly elevated in lung cancer compared to controls. MUC1 [P15941] is a membrane-bound mucin of 122 kDa molecular weight with several interacting isozymes, polymorphic tandem repeats, and an extensively O-glycosylated core protein [49]. *In vitro *studies suggested that MUC1 reduces E-cadherin-mediated cell-cell adhesion by steric hindrance, which increases metastatic ability [50]. High MUC1 levels also reduces the integrin-mediated cell adhesion to the extracellular matrix [51]. The clinical importance of the MUC1 glycoprotein, however, is not clear. Previous studies have reported that MUC1 was developmentally regulated and aberrantly expressed by carcinomas, and a high level of MUC1 mRNA expression in adenocarcinoma has been associated with poor prognosis [52-58]. MUC1 was also found to be up-regulated in non-small-cell lung cancer [59-61]. MUC1 is shed into the blood stream and thus has a potential as a tumor marker, as demonstrated in breast cancer [62-64]. Consistent with this finding, we observed higher MUC1 expression levels in the sera of lung cancer patients than in either healthy subjects or patients with COPD. Additionally, MUC1 expression levels did not show significant correlation with CRP, SAA, or AAT, suggesting that the increased MUC1 levels might be due to a different biological process. Interestingly, MUC1 serum levels in breast cancer patients were not concordant with the levels observed in tumor tissues by immunohistochemistry [64,65], so the increased serum MUC1 expression may correspond to a specific isoform expressed by cancer cells. Thus, expression levels of the different MUC1 isoforms and their epitopes may need to be evaluated to fully explain the increased levels in serum of lung cancer patients.

Other acute-phase reactant serum proteins that have been reported as significantly elevated in certain cancers were not increased in this study of sera from lung cancer patients. Most notably, the alpha sub-unit of haptoglobin (MW 11.7 kDa) and isoforms of the haptoglobin-1 precursor (HAP1) have been reported to be increased in serum of patients with ovarian and other gynecologic cancers [66,67].

## Conclusion

Our results suggest that a distinctive serum protein profile involving relatively abundant proteins may be observed in cancer patients relative to healthy subjects or patients with chronic disease. It is therefore likely that distinctive mass peak profiles observed by mass spectrometry in cancer sera relative to control and that may be predictive of outcome include a significant component related to host response to tumors and acute phase reactants. The extent to which such indicators of host response have clinical utility as a group, together with other tumor biomarkers remains to be determined. The use of antibody microarrays directed against a broad range of serum and lung tumor proteins would have utility for elucidating those proteins with the greatest diagnostic utility.

## Competing interests

The author(s) declare that they have no competing interests.

## Authors' contributions

W-MG carried out the antibody microarray studies, participated in the statistical treatment of the data and drafted the manuscript. RK performed the statistical analysis of the data and in drafting the manuscript. RPO participated in the antibody microarray studies. DEM participated in the design of the study, helped coordinate serum acquisition/usage and in drafting the manuscript. JQ carried out the independent validation of CRP and SAA quantitation. AKG and WNR participated in the acquisition of the COPD serum. DEB participated in the acquisition of both the normal and control serum. GSO participated in the design of the study and helped to draft the manuscript. BBH participated in the design of the study and its coordination, and helped to draft the manuscript. SMH conceived of the study, participated in its design, and helped to draft the manuscript. All authors read and approved the final manuscript.

## Pre-publication history

The pre-publication history for this paper can be accessed here:



## Figures and Tables

**Table 1:** Results for 7 antibodies showing significant differences between both lung tumor patients vs. normal controls and lung tumor patients vs. COPD patients.

	Fold difference in means		P-values from 1-way ANOVA		P-value from 2-sample T-test
Antibody	Tumor / Normal	Tumor / COPD	Tumor vs. Normal	Tumor vs. COPD	Tumor vs. Others

CRP	13.6	13.0	1.1 × 10^-9^	2.2 × 10^-10^	4.1 × 10^-12^
SAA	1.99	2.15	1.8 × 10^-7^	1.7 × 10^-9^	2.5 × 10^-10^
MUC1	1.30	1.42	3.3 × 10^-3^	4.3 × 10^-5^	5.3 × 10^-5^
AAT (1)	1.34	1.35	9.1 × 10^-4^	3.1 × 10^-4^	7.9 × 10^-5^
AAT (2)	1.42	1.33	1.1 × 10^-3^	3.7 × 10^-3^	5.2 × 10^-4^
Transferrin	0.73	0.71	2.7 × 10^-4^	2.3 × 10^-5^	7.2 × 10^-6^
Gelsolin	0.77	0.77	5.8 × 10^-3^	4.7 × 10^-3^	1.4 × 10^-3^

**Table 2:** Correlation between CRP, SAA, AAT, MUC1, and Transferrin protein expression in the serum of lung tumor patients.

	SAA		AAT (1)		AAT (2)		MUC1		Transferrin	
	r	p value	r	p value	r	p value	r	p value	r	p value

CRP	0.60	0.0019	0.62	0.0012	0.61	0.0015	0.19	0.37	-0.175	0.41
SAA			0.48	0.019	0.41	0.049	-0.07	0.74	-0.121	0.57
AAT (1)					0.72	6.63 × 10^-5^	-0.02	0.94	0.012	0.96
AAT (2)							0.17	0.44	0.042	0.85
MUC1									0.226	0.29
Transferrin										
